# Central and peripheral excitability in restless limbs syndrome

**DOI:** 10.1093/braincomms/fcaf506

**Published:** 2025-12-24

**Authors:** Amedeo De Grado, Gaia Fanella, James Howells, Benjamin Yamin Ali Khan, Anna Bystrup Jacobsen, Bülent Cengiz, Gintaute Samusyte, Marit Otto, Paola Lanteri, Ambra Stefani, Hatice Tankisi

**Affiliations:** Neurophysiology Unit, Fondazione IRCCS Istituto Neurologico ‘Carlo Besta’, Milan, Italy; Università Degli Studi di Milano, Milano, Italy; Unit of Rare Neurological Diseases, Fondazione IRCCS Istituto Neurologico ‘Carlo Besta’, Milan 20133, Italy; Aldo Ravelli Center for Neurotechnology and Experimental Brain Therapeutics, Department of Health Sciences, University of Milan, Milan, Italy; Department of Clinical Neurophysiology, Aarhus University Hospital, Aarhus, Denmark; Department of Neurology, Fondazione IRCCS San Gerardo dei Tintori, School of Medicine and Surgery, Milan Center for Neuroscience, University of Milano-Bicocca, Monza, Italy; Central Clinical School, Faculty of Medicine and Health, University of Sydney, Sydney, Australia; Department of Clinical Neurophysiology, Aarhus University Hospital, Aarhus, Denmark; Department of Clinical Neurophysiology, Aarhus University Hospital, Aarhus, Denmark; Department of Neurology, Gazi University Faculty of Medicine, Beşevler 06500, Ankara, Turkey; Neuroscience and Neurotechnology Centre of Excellence, Gazi University, Ankara, Turkey; Department of Neurology, Medical Academy, Lithuanian University of Health Sciences, Kaunas, Lithuania; Department of Clinical Neurophysiology, Aarhus University Hospital, Aarhus, Denmark; Neurophysiology Unit, Fondazione IRCCS Istituto Neurologico ‘Carlo Besta’, Milan, Italy; Università Degli Studi di Milano, Milano, Italy; Center for Sleep Medicine, Department of Neurology, Medical University of Innsbruck, Innsbruck, Austria; Department of Clinical Neurophysiology, Aarhus University Hospital, Aarhus, Denmark

**Keywords:** axonal excitability, clinical neurophysiology, restless limbs syndrome, spinal excitability, threshold-tracking transcranial magnetic stimulation

## Abstract

Restless limbs syndrome (RLS) is a neurological disorder characterized by an uncontrollable urge to move the limbs. Although it affects up to 10% of the general population, its underlying mechanisms remain poorly understood. Neurophysiological excitability testing may help elucidate mechanisms related to sensorimotor integration, axonal ion channel dysfunction and impaired neural inhibition. This study aimed to assess both CNS and PNS function by examining cortical, spinal and peripheral nerve excitability within the same individuals for the first time. To investigate potential widespread excitability changes in RLS, we specifically analysed hand muscles, offering new insights into the extent of neural involvement beyond the lower limbs. The study included 56 RLS patients, divided into treated and untreated groups, along with 32 healthy controls. Notably, none of the patients experienced symptoms in their hands. Cortical excitability was assessed via threshold-tracking transcranial magnetic stimulation (TMS) to evaluate intracortical inhibition and facilitation. Sensory-motor integration was measured via long-latency reflexes (LLRs), while spinal cord excitability was assessed using F-waves, H-reflexes and RIII-reflexes. Axonal excitability was examined using the extended TRONDNF protocol. TMS revealed a significant reduction in short-interval intracortical inhibition (SICI) in patients, particularly at inter-stimulus intervals (ISIs) of 2.5 and 3 ms. When averaging across ISIs from 1 to 7 ms, patients on medication exhibited significantly less inhibition compared to healthy controls. Long-interval intracortical inhibition (LICI) was also reduced at ISIs of 150 and 200 ms, while facilitation parameters remained within normal ranges. Patients exhibited increased amplitude of the second component of the LLR recorded from the abductor pollicis brevis, whereas RIII reflex measurements showed no significant differences. Axonal excitability testing revealed a graded increase in hyperpolarization-activated currents in patients with more severe symptoms. The observed reductions in SICI and LICI suggest impaired intracortical inhibition in the M1 hand area, offering indirect evidence of cortical dysfunction in regions clinically unaffected by the disease. The increased LLR amplitudes further indicate altered sensorimotor integration at the cortical level, whereas the absence of significant changes in RIII reflexes suggests that segmental spinal dysfunction within the pain pathway of the upper limbs is unlikely. Finally, axonal excitability findings point to a potential role of hyperpolarization-activated currents in either contributing to or predisposing individuals to RLS symptoms.

## Teaser

De Grado *et al*. report that patients with restless limbs syndrome exhibit altered cortical excitability, impaired sensorimotor integration and abnormal nerve excitability even in clinically unaffected body regions. These findings suggest widespread cortical dysfunction and implicate ion channel abnormalities as potential contributors to the pathophysiology of this common disorder.

## Graphical Abstract

**Graphical Abstract:**
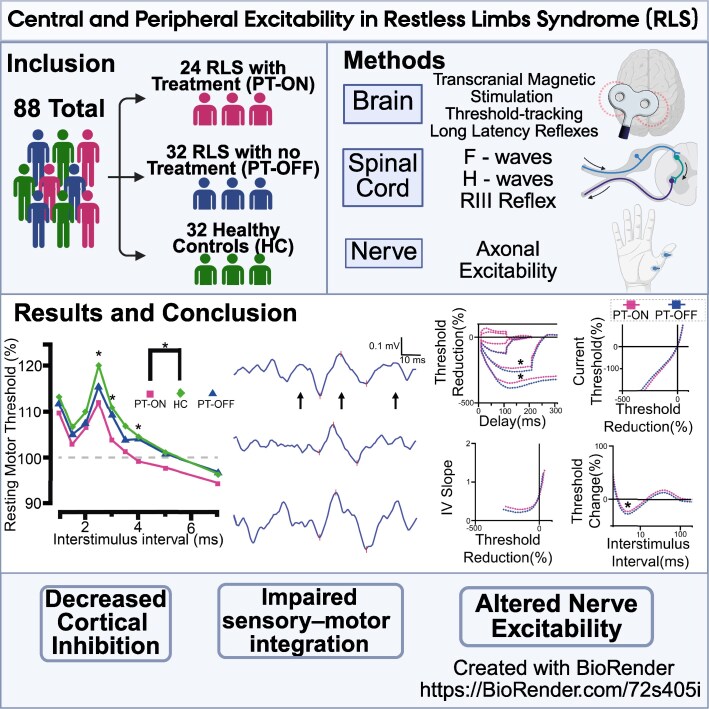

## Introduction

Restless limbs syndrome (RLS), formerly known as restless legs syndrome, is a neurological sensory-motor disorder characterized by an irresistible urge to move the limbs, particularly during periods of inactivity (in the evening or night), often accompanied by unpleasant sensations that are transiently alleviated by movement.^1^ RLS has a strong familial association and is linked to various neurological and systemic conditions, including iron deficiency, Parkinson’s disease, radiculopathies and pregnancy.^2^ Its estimated prevalence is approximately 10% among adults in European and North American populations, with a notable impact on quality of life.^3^ Beyond its effects on daily activities, RLS-related sleep disturbances contribute to significant functional impairment, reducing work productivity and increasing the risk of depression and anxiety.^4^

Despite ongoing research, the precise pathophysiology of RLS remains incompletely understood. Current hypotheses suggest a multifactorial aetiology involving genetic predisposition,^5^ brain iron deficiency,^6^ GABAergic dysfunction,^7^ dopaminergic dysregulation^8^ and central sensitization of A-delta fibre high-threshold mechanoreceptor inputs.^9,10^ Additionally, increased excitability within spinal cord circuits and motoneurons/motor axons has been implicated.^11-14^ Neurophysiological studies indicate a widespread decrease in CNS motor inhibition while the involvement of the PNS remains debated, although recent findings suggest that axonal excitability alterations may contribute to disease pathology.^15^

CNS involvement in RLS has been investigated using transcranial magnetic stimulation (TMS), a non-invasive technique widely used to assess cortical and subcortical networks through single, paired or multiple-pulse protocols.^16-20^ While a detailed discussion of paired-pulse paradigms is beyond the scope of this paper, their physiological significance has been extensively studied.^20-26^ Briefly speaking, cortical inhibition can be evaluated using paired-pulse TMS with interstimulus intervals (ISIs) of 1–6 ms for short-interval intracortical inhibition (SICI), which is mediated by GABA_A_ receptors,^20,27,28^ and ISIs of ∼100–300 ms for long-interval intracortical inhibition (LICI), which is mediated by GABA_B_ receptors.^20,22,29,30^ Cortical facilitation can be evaluated adjusting the intensity of the conditioning and test stimuli and using either ISIs of 10–15 ms for intracortical facilitation (ICF), which is likely involving the NMDA pathway,^20^ or discrete ISIs of 1.1–1.5, 2.3–3.0 and 4.1–4.5 ms for short intracortical facilitation (SICF).^20,24,31^ One of the most consistent TMS findings in RLS is a reduction in SICI,^32,33^ which seems to correlate with disease severity and tends to normalize, at least partially, following treatment with dopamine agonists.^34-36^ This reduction in SICI has been observed in both upper and lower limb muscles, therefore suggesting a widespread decrease in CNS motor inhibition.^33,37-40^ Notably, these changes are not merely a consequence of sleep deprivation,^41,42^ nor are they influenced by sleep deprivation, as SICI measurements remain unaffected by lack of sleep.^43^ On the other hand, the role of LICI and ICF alterations in RLS remains unclear, as studies have reported inconsistent findings, likely due to methodological differences, variations in medication status and outdated diagnostic criteria.^32,34,36,37,39,40,44-47^ Finally, while SICF has been explored in other neurological disorders, its role in RLS remains uninvestigated.

Another key aspect of RLS pathophysiology appears to involve spinal cord dysfunction, in the form of an imbalance between inhibitory and excitatory mechanisms within motor pathways.^48^ Neurophysiological studies suggest increased spinal cord excitability, as shown by investigations employing the nociceptive flexion reflex (NFR), a polysynaptic and multi-segmental spinal reflex. The NFR typically present as a double burst: an early, inconsistent component known as the RII reflex, and a later, larger, and more stable component called the RIII reflex. In patients with RLS, this reflex is altered in the lower limbs, particularly during sleep, when spinal reflexes are normally more strongly inhibited.^11,49^ Moreover, RLS patients exhibit heightened sensitivity to mechanical pinprick stimuli, suggesting a central sensitization process that may contribute to the characteristic discomfort and urge to move the limbs. Whether these findings reflect a primary excitability abnormality within the spinal cord or result from reduced cortical inhibition remains under active investigation.^10-12,50^ For a comprehensive review of spinal cord involvement in RLS, see Paulus and Schomburg.^48^

Finally, PNS involvement in RLS has traditionally been considered secondary. However, recent studies using axonal excitability testing suggest a potential peripheral contribution. In fact, while standard nerve conduction studies are typically unremarkable, unless a coexisting polyneuropathy is present,^50^ advanced electrophysiological techniques have revealed increased activity of hyperpolarization-activated cyclic nucleotide-gated (HCN) channels in motor, but not sensory, axons of the median nerve. This finding suggests a broader alteration in motoneuron excitability,^15^ supporting the hypothesis that RLS involves both CNS and PNS dysfunction.

Building on these findings, our study aims to provide a comprehensive understanding of the neural mechanisms underlying RLS by integrating multiple neurophysiological assessments within a single session. Specifically, the combined use of TMS, spinal reflex testing and axonal excitability measurements enables a detailed evaluation of both central and peripheral contributions to disease pathophysiology. Moreover, by examining the upper limbs, we aim to demonstrate that excitability abnormalities extend beyond clinically affected regions, supporting the hypothesis of widespread dysfunction in both the CNS and PNS. Identifying these alterations may yield valuable biomarkers for RLS diagnosis and treatment monitoring, ultimately improving clinical management and guiding therapeutic strategies.

## Materials and methods

We recruited 56 patients with RLS and 32 healthy controls (HC) matched for age and sex. A comprehensive neurological examination assessing strength, deep tendon reflexes, and all sensory modalities was conducted to exclude inclusion of patients or HCs with polyneuropathy. All subjects were ≥18 years old and provided informed consent, and the study was carried out in accordance with the Declaration of Helsinki II. The Regional Scientific Ethical Committee (Case number: 1-10-72-177-22) approved the study.

Exclusion criteria for both groups included: (1) a history of CNS or PNS disease apart from RLS; (2) comorbid iron-deficiency anaemia, chronic renal failure and severe heart failure; (3) pregnancy; (4) history of epilepsy; (5) presence of a pacemaker or intracranial implants; and (6) use of medications known to affect the CNS (other than pramipexole or ropinirole used to treat RLS).

RLS diagnosis was established following the current International RLS Study Group (IRLSSG) criteria.^1^ Patients on medication were instructed to gradually taper and discontinue their treatment for at least 5 days before the study, whenever possible. Alternatively, they were advised to take the minimum effective dose necessary for symptom control. Of the 26 patients on medication, only two were able to completely discontinue the treatment and were therefore included in the OFF-medication group. Four others reduced their dosage to the lowest effective level and were therefore retained in the ON-medication group. The remaining patients in the OFF-medication group reported never having taken medication for RLS. All participants were provided with an information sheet listing substances known to affect CNS excitability (i.e. caffeine and other common stimulants) and were instructed to avoid their consumption in the 24 h preceding the study. Due to patients’ refusal to reduce their medication (owing to symptom worsening) and based on findings from three key studies that established the ‘entry-level’ dose for dopamine-agonists,^51-53^ the medication dosages in use at the time of study enrolment were taken as a proxy for disease severity. Axonal excitability data were accordingly categorized into three groups: (1) patients not on medication (PT-OFF); (2) patients on a low-dose regimen (less than three times the typical LEDD starting dose of 13, PT-low); and (3) patients on a medium-to-high dose (more than three times the typical LEDD starting dose of 13, PT-medium-high).

A warming lamp was used to maintain a temperature consistently above 32°C during neurophysiological measurements. For all recordings, Ag/AgCl surface electrodes were placed over the muscle in a belly-tendon configuration. For the abductor pollicis brevis (APB), the reference electrode was placed on the proximal phalanx of the thumb and the ground electrode on the dorsum of the hand; for the flexor carpi radialis (FCR), the reference electrode was placed on the ulnar styloid, with the ground electrode on the volar forearm. All recordings were performed between 8am and 4pm. Notably, no patients exhibited or reported symptoms involving the hands.

### Transcranial magnetic stimulation (TMS)

Subjects were comfortably seated in an armchair. TMS was conducted using two Magstim 200^2^ stimulators connected in a BiStim configuration (Magstim Co. Ltd, Whitland, Wales, UK) to a figure-of-eight coil. Stimulus delivery and data acquisition were controlled by QTRACW (software ©UCL, distributed by Digitimer Ltd.) using QTMSG-12 recording protocols (QTMS Science). Magnetic stimuli were delivered at 4.5- or 5-s intervals.

The coil was placed flat on the head over the left motor cortex, at an angle of approximately 45° to the sagittal plane, inducing a current in the brain roughly perpendicular to the central sulcus, flowing from posterior to anterior. Optimal coil placement was determined by recording motor evoked potentials (MEPs) while moving the coil over the head; the position yielding the highest peak-to-peak amplitude of the MEP (hot spot) was marked with a semi-permanent pen on a cap worn by the subject to ensure accurate coil positioning throughout the testing. The subjects were kept awake throughout the examination.

Due to its possible higher sensitivity in detecting cortical excitability impairments in ALS compared with conventional amplitude-based approaches,^54,55^ we decided to employ parallel threshold-tracking TMS in this study.^28,56-59^

The resting motor threshold (rMT200) was defined as the stimulus intensity (as a % of maximum stimulator output) required to track a target MEP response amplitude of 0.2 mV, which lies in the middle of the steepest portion of the TMS stimulus-response curve.^28,60^ Adequate measures of threshold were determined as either: falling within the target response 200 µV ± 20% (on a log scale, i.e. from 160 to 250 µV); or if successive responses bracketed the target response. The resting motor threshold was determined using the ‘4->2->1’ tracking and logarithmic regression method rule as previously described.^61^ Subsequently, changes in the test stimulus intensity required to maintain the MEP response at this preset threshold, when preceded by either sub-threshold or supra-threshold conditioning stimuli, were measured (the ‘threshold-hunting’ paradigm).^20,56^

We evaluated 9 ISIs for SICI (1, 1.5, 2, 2.5, 3, 3.5, 4, 5, 7 ms), 5 for ICF (10, 15, 20, 25, 30 ms), 14 for SICF (1, 1.3, 1.6, 1.9, 2.2, 2.5, 2.8, 3.1, 3.4, 3.7, 4, 4.3, 4.6, 4.9 ms) and 6 for LICI (50, 100, 150, 200, 250 and 300 ms). For assessments of SICI, SICF and ICF, we tracked the stimulus threshold at each ISI using 10 paired-pulse stimuli. Each subsequent stimulus threshold was adjusted in proportion to the error between the last and target responses with maximum stimulus step sizes reducing from 6% to 2%. The ISI order was pseudo-randomized so to minimize potential biases associated with the ‘serial’ method as described by Tankisi *et al.*^61^

We also recorded the CSP during maximal muscle contraction using five different stimulation intensities expressed as a percentage of the rMT (80%, 100%, 120%, 140% and 160%) and obtained three traces for each intensity. We defined CSP duration as ‘the interval between the CSP onset and the last resumption of electromyographic (EMG) activity following the cortical silent period’ and CSP onset as ‘the interval between the MEP onset and the start of the cortical silent period’.

All TMS measurements are expressed as a percentage of the maximal stimulus output ± standard error.

### Spinal cord excitability and LLRs

Patients were seated comfortably with one arm resting on an armrest. All the recordings were performed using a Keypoint EMG machine. Compound muscle action potential (CMAP) amplitudes and H-reflexes were recorded from APB and FCR by stimulating the median nerve at the wrist or at the forearm. H-reflexes were recorded during a steady muscle contraction at 20–30% of the maximal strength. F-waves were also recorded from APB.

The nociceptive flexion reflex was recorded in response to electrical stimulation of the index finger using ring electrodes. Five 1 ms pulses with a 2 ms ISI were delivered to the finger, with responses recorded from APB and FCR muscles using surface electrodes. RIII-reflex threshold was determined by adjusting the stimulus intensity until a stable reflex response from the FCR was obtained. Three stimuli were then delivered at irregular intervals over 15 s at intensities of ×2 and ×4 above baseline RIII threshold, with a 10-mA upper limit to reduce discomfort. Parameters measured included maximal amplitude, minimal latency, response duration and area-under-the-curve. Markers were set by a blinded operator (H.T.) based on earlier RIII studies performed on the upper limbs.^62^ To determine the RIII response parameters, the operator examined all the curves for the ×4 intensity, selecting the one with the highest amplitude and the clearest onset. The highest positive RIII amplitude was determined after superimposing the curves within a window of 50–200 ms after stimulus onset. The statistical analysis was then performed by another operator (A.D.G.).

Additionally, to further explore spinal cord afferents, sensory-motor cortical integration and motor output, we also included a study of long latency reflexes (LLRs) from the hands.^63^ The LLR protocol involved recording the surface EMG signal from APB and FCR during a steady muscle contraction at 20–30% of maximal contraction by stimulating the median nerve at the wrist or forearm.^64^ Stimulation intensity was set at the amplitude yielding the highest H-reflex amplitude (assessed visually), with a pulse width of 200 µs and a frequency of 3 Hz.^64^ We averaged at least 200 traces three times. Amplitudes and latencies were calculated for each LLR wave, resolving doubts using Deuschl *et al.*'s equation.^65^

### Axonal excitability testing with threshold tracking

The recording setup mirrored that used for the TMS examination. We employed two round, non-polarizable Ag/AgCl adhesive electrodes; the cathode was positioned over the median nerve near the wrist, while the anode was placed on the radial border of the forearm, maintaining a distance of at least 10 cm from the cathode. The protocol was controlled by the QtracS software running the TRONDNF protocol with extended hyperpolarizing threshold electrotonus (70% and 100%) to probe hyperpolarization-activated cyclic nucleotide gated channels.^66,67^

Briefly, the program recorded a stimulus response curve, and the response target was set to 40% of the maximal response. The amplitude of the stimulus current corresponding to this target is referred to as the ‘threshold’.^68^ Two sequential stimuli were then applied: a ‘conditioning stimulus’ followed by a ‘test stimulus’. In threshold tracking, if the test response falls below the tracking target, the intensity of the subsequent stimulus is increased; conversely, if the response exceeds the target, the intensity of the subsequent stimulus is reduced. This adaptive adjustment helps maintain a consistent test potential size across different manoeuvres, preventing the so-called ‘floor’ and ‘ceiling’ effects, also enabling the study of axons of similar size despite changes in their excitability.^66,68-71^ Variations in stimulus duration and strength of both stimuli form the basis for studying channel kinetics, while changes in threshold values are mathematically modelled to interpret abnormalities in ion currents and channel function at the nodal–paranodal region. A detailed description of the TRONDNF protocol and the techniques employed can be found in Bostock *et al.*^66^ and in Burke *et al.*^70^ Axonal excitability has been used to investigate pathophysiological mechanisms underpinning several different diseases such as spinal muscular atrophy,^72^ episodic ataxia type 1,^73^ oxaliplatin-induced neurotoxicity,^74^ multifocal motor neuropathy^75^ and ALS.^76^

Currently, there are no known/published effects on peripheral excitability of the RLS medications used by our patients.

### Data analysis

QtracP, a component of the QTRACW software package, was used for all analysis, including statistical testing, plot generation and CSP measurement. Data normality was evaluated using the Lilliefors test. Variables that satisfied normality assumption were analysed using a two-tailed independent-samples Student’s *t*-test, whereas non-normally distributed variables were evaluated using the Mann–Whitney U test. Parametric data are reported as mean ± SEM, and non-parametric data as median [Q1, Q3]. The study was designed around predefined, hypothesis-driven pairwise comparisons (e.g. patients on medication versus healthy controls; patients off medication versus healthy controls; all patients versus healthy controls), rather than simultaneous evaluation of multiple groups. Because each contrast involved only two independent groups, the independent-samples *t*-test was the appropriate parametric method. An ANOVA was not deemed necessary, as no omnibus multi-group comparisons were conducted. When normality assumptions were not met, the Mann–Whitney U test was applied as the robust non-parametric alternative. A *P*-value < 0.05 was considered statistically significant.

## Results

**Figure 1:**
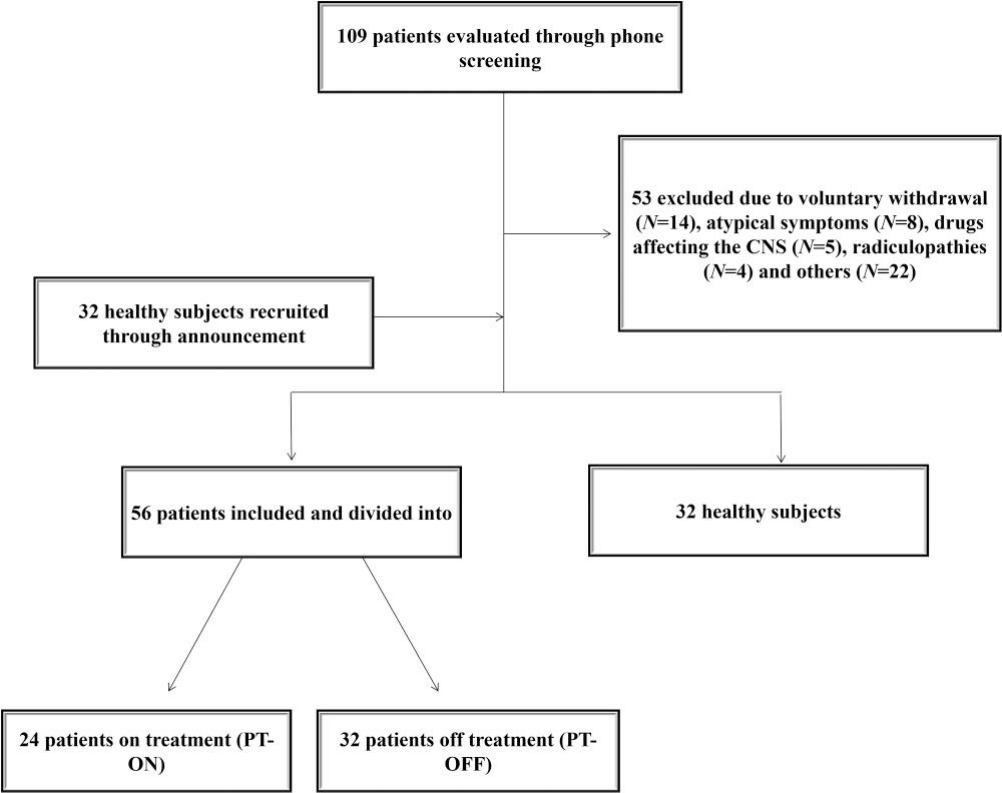
Patients and healthy controls evaluation and selection process.

We screened 109 patients and excluded 53 subjects. The most common causes of exclusion were voluntary withdrawal from the study (*N* = 14), symptoms not fulfilling the diagnostic criteria (*N* = 8), CNS-affecting drug use other than those used for RLS (*N* = 5) and lower limb radiculopathies (*N* = 4) (see Fig. 1).

We included 56 patients and 32 healthy controls, dividing the patients’ group into those on medications (PT-ON, *N* = 24) and those off medications (PT-OFF, *N* = 32). The mean age was 47.2 ± 12.7 for the PT-TOT group, 51 ± 11.2 for the PT-ON group, 51 ± 3.3 for the PT-ON low group, 52.4 ± 2.6 for the PT-ON med-high group, 43.9 ± 12.9 for the PT-OFF group and 47.7 ± 13.5 for the healthy controls (HCs). Demographic data are provided in Table 1. There were no statistically significant differences in age, sex or temperature between the cohorts. Almost all subjects completed the entire protocol, with only six exclusions from LICI due to the ceiling effect and six patients from axonal excitability due to high skin impedance or temperatures ≤ 32°C despite the warming lamp. Regarding CSP, some patients were unable to tolerate stimulation at the highest intensities, resulting in a variable number of available data points per intensity level. For the HC group, data were obtained from the following number of participants: N80% = 29, N100% = 31, N120% = 30, N140% = 27 and N160% = 20. For the PT group, the numbers were: N80% = 27, N100% = 27, N120% = 28, N140% = 23 and N160% = 16. For further details about LLRs and RIII, refer to Table 2.

**Table 1:** Pooled demographic data

Group	No. of subjects	Age (years)	Sex	Mean LEDD	Mean disease duration (years)
Healthy Controls	32	47.7 ± 13.2	59% F	NA	NA
PT-TOT	56	47.2 ± 12.7	71.4% F	22.3	19.3 ± 1.9
PT-OFF	32	43.9 ± 12.9	69% F	NA	15.8 ± 2.3
PT-ON	24	51 ± 11.2	75% F	57.3	23.9 ± 3.1
PT-ON low	15	51 ± 3.3	80% F	21.8	21.5 ± 4.3
PT-ON med-high	9	52.4 ± 2.6	66% F	97.3	28 ± 4

F = female, LEDD = levodopa equivalent daily dose, low = low dose of LEDD, med-high = medium-to-high dose of LEDD, PT-OFF = patients off medication, PT-ON = patient on medication, PT-TOT = total of patients.

**Table 2:** Long-latency reflexes and spinal excitability parameters

Parameters	HC	PT-TOT	PT-ON	PT-OFF
APB—LLR I amplitude	36.4 [24.62; 60.92] (32)	49.9 [31.90; 79.90] (51)	46.2 [36.20; 80] (21)	53.15 [30.78;76.02] (30)
APB—LLR II amplitude	61.5 [40; 91.15] (32)	96.2 [59.45; 157] (51)	81.2 [45.80; 201.7] (21)	104.8 [62.73; 147] (30)
APB—LLR III amplitude	25.3 [17.25; 37.25] (27)	27.95 [17.60; 41.15] (44)	34.5 [17.70; 64.70] (17)	26.9 [19.05; 39.35] (27)
FCR—LLR I amplitude	11.80 [7.50; 18.50] (25)	13.85 [9.40; 18.55] (34)	16.1 [9.10; 21.90] (13)	13.30 [10.30; 15.20] (21)
FCR—LLR II amplitude	11 [10.1; 18.4] (25)	15.85 [9.32; 23.62] (38)	17 [9.85; 25.6] (19)	14.70 [9.35; 18.25] (19)
FCR—LLR III amplitude	10.70 [6.90; 16.40] (17)	16.90 [11.48; 19.23] (24)	14.05 [11.02; 19.15] (14)	17.50 [14.8; 18.77] (10)
APB—RIII onset latency	80.49 ± 4.70 (16)	80.61 ± 2.45 (36)	81.49 ± 3.74 (15)	80.02 ± 3.31 (21)
APB—RIII duration	73.04 ± 5.78 (16)	72.5 ± 3.37 (35)	74.95 ± 7.07 (14)	70.86 ± 3.20 (21)
APB—RIII amplitude	0.079 [0.03; 0.15] (16)	0.078 [0.05; 0.12] (35)	0.079 [0.05; 0.11] (14)	0.07 [0.05; 0.18] (21)
APB—RIII area	1.08 [0.80; 2.15] (16)	1.15 [0.70; 1.98] (36)	1.11 [0.67; 2.05] (15)	1.18 [0.70;1.91] (21)
FCR—RIII onset latency	71.25[67.5; 83.4] (26)	73 [65.2; 80.2] (53)	71.95 [65.43; 85.82] (24)	73.6 [65.2; 77.5] (29)
FCR—RIII duration	104.6 ± 4 (26)	98.91 ± 2.877 (53)	100.8 ± 4.357 (24)	97.32 ± 3.871 (29)
FCR—RIII amplitude	0.22 [0.11; 0.46] (26)	0.2 [0.11;0.6] (53)	0.21 [0.10; 0.52] (24)	0.18 [0.12; 0.62] (29)
FCR—RIII area	4.65 [1.928; 7.15] (26)	4.5 [1.895; 12.7] (52)	4.6 [2.25; 11] (23)	4.2 [1.67; 17.7] (29)

Parametric variables are expressed as mean ± standard error. Non-parametric variables are expressed as median followed by squared brackets displaying upper and lower quartiles values. The number in the round brackets represents the sample size. APB = abductor pollicis brevis, FCR = flexor carpi radialis, HC = healthy controls, LLR I = long latency reflex I, LLR II = long latency reflex II, LLR III = long latency reflex III, PT-OFF = patients off medication, PT-ON = patient on medication, PT-TOT = total of patients.

### TMS

The rMT200 was 58.14 ± 1.78 in the HCs group, 60.87 ± 1.6 in the PT-TOT group, 60.6 ± 1.88 in the PT-ON group and 61.2 ± 2.8 in the PT-OFF group. Statistical analysis did not disclose any significant difference in rMT200 among the groups.

**Figure 2:**
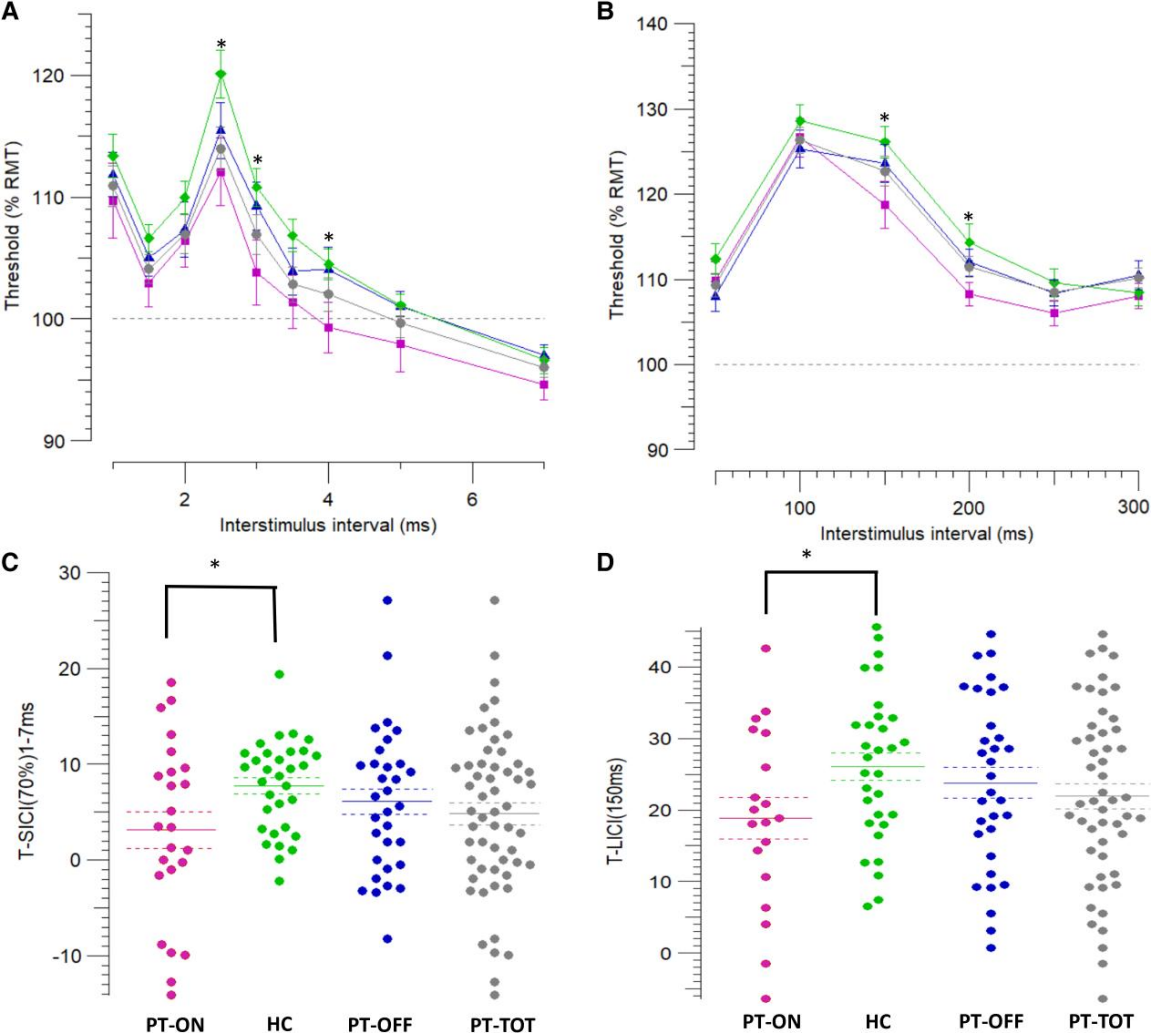
**Parallel threshold-tracking short-interval intracortical inhibition (SICI; A–C) and long-interval intracortical inhibition (LICI; B–D) data.** (**A**) Both the PT-TOT and PT-ON groups show reduced inhibition compared to HC, particularly at the 2.5 ms ISI. For each group, SICI values between 1 and 7 ms were averaged within participants; each point reflects this group-level mean ± SE, with upward deflections indicating greater inhibition. PT-TOT: *N* = 56, PT-ON: *N* = 24, PT-OFF: *N* = 32, HC: *N* = 32. (**B**) The PT-ON group demonstrates significantly reduced inhibition at 150 and 200 ms compared with HC. PT-TOT: *N* = 51, PT-ON: *N* = 19, PT-OFF: *N* = 32, HC: *N* = 31. (**C**) Distribution of individual mean SICI values (1–7 ms ISI) showing reduced inhibition in PT-ON compared to HC. Central bars and dashed lines indicate mean ± SE. *N* = 56, PT-ON: *N* = 24, PT-OFF: *N* = 32, HC: *N* = 32. (**D**) Individual LICI values at 150 ms, with reduced inhibition observed in PT-ON compared to HC. Central bars and dashed lines indicate mean ± SE. PT-TOT: *N* = 51; PT-ON: *N* = 19; PT-OFF: *N* = 32; HC: *N* = 31. Squares: patients on medication (PT-ON); Diamonds: healthy controls (HC); Triangles: patients off medication (PT-OFF); Dots: all patients (PT-TOT). ISI = interstimulus interval, RMT = resting motor threshold, SE = standard error. *=*P* < 0.05. Student’s *t*-test was used.

Regarding SICI, the 2.5 ms ISI was the most affected, with the PT-TOT group exhibiting significantly reduced inhibition compared to HCs (13.99 ± 1.77% versus 20.09 ± 1.96%, *P* = 0.02). Subgroup analysis indicated that this difference was primarily driven by the PT-ON group (12.06 ± 2.8, *P* = 0.01) (see Fig. 2). The second most affected ISI was 3.0 ms (3.81 ± 2.7 versus 10.81 ± 1.56, *P* = 0.02), with PT-ON showing almost no inhibition compared to HCs. Moreover, when averaging across ISIs from 1 to 7 ms, PT-ON demonstrated significantly less inhibition than HCs (3.12 ± 1.9% versus 7.76 ± 3.2%, *P* = 0.01).

Similarly, the PT-ON group exhibited significantly reduced inhibition at both 150 ms (18.85 ± 2.9 versus 26.09 ± 1.89, *P* = 0.03) and 200 ms ISIs (8.35 ± 1.47 versus 14.41 ± 2.15, *P* = 0.04) compared to HCs. No statistically significant differences were observed in SICF, ICF (as outlined in Supplementary Fig. 1) or in any of the CSP-related parameters across the subgroups (data not shown).

### Spinal cord and sensory-motor integration

**Figure 3:**
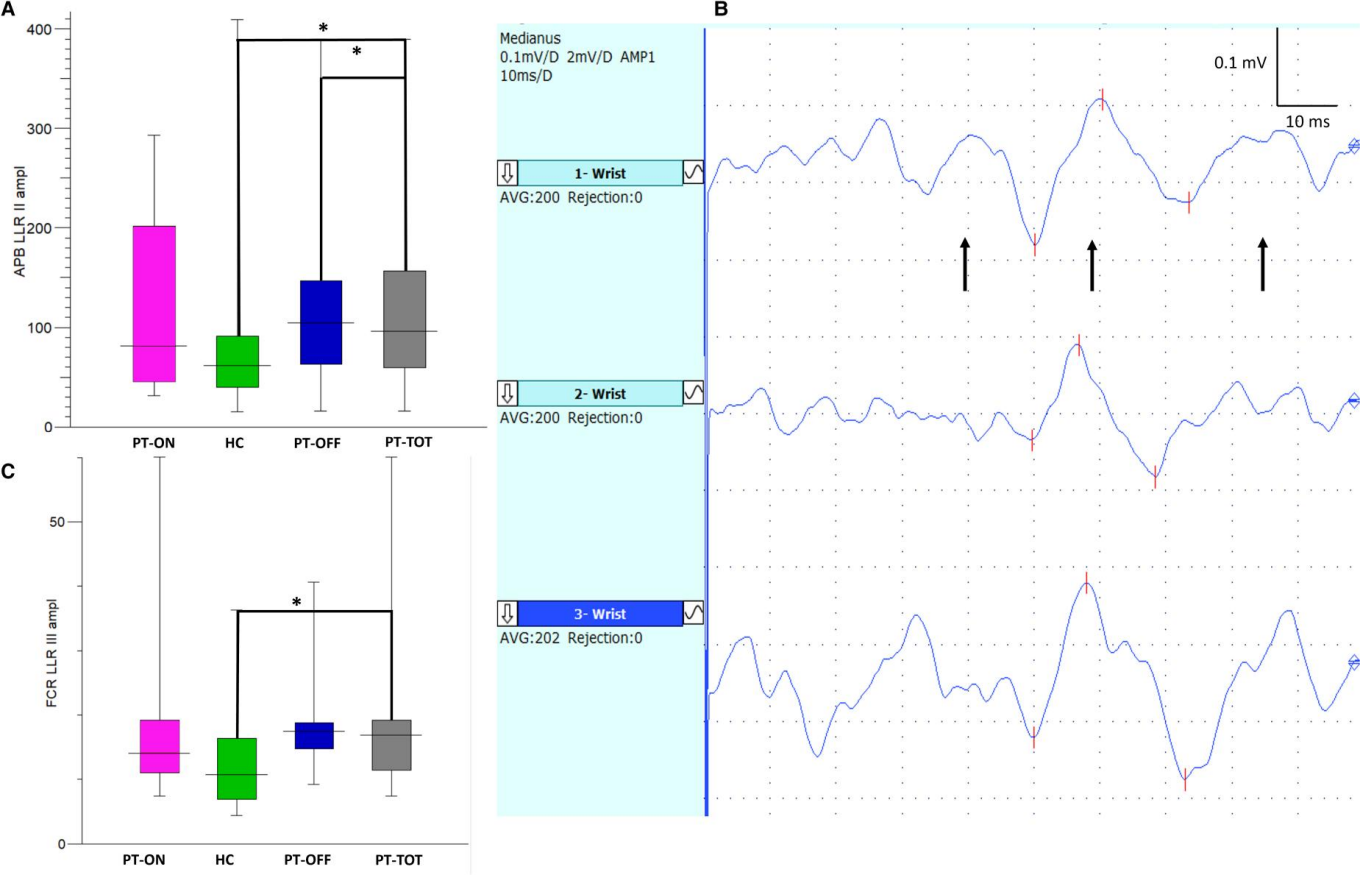
**Long latency reflexes (LLRs).** (**A**) LLRII amplitude from the APB: both PT-TOT (*N* = 51) and PT-OFF (*N* = 30) exhibit a significant increase in the LLRII amplitude response compared to HC (*N* = 32). Mann–Whitney U test was used. (**B**) An example of long latency reflexes recording is presented, with black arrows indicating the LLRI, LLRII and LLRIII waves in succession. We averaged 200 traces three times and then calculated the mean amplitude and mean latency for each wave. (**C**) LLRIII amplitude from FCR: PT-TOT (*N* = 24) group shows an increase in the LLRIII amplitude compared to HC (*N* = 17). Mann–Whitney U test was used. Refer to the text for interpretation. APB = abductor pollicis brevis, FCR = flexor carpi radialis, HC = healthy controls, LLRII = second component of the long latency reflexes, LLRIII = third component of the long latency reflexes, PT-OFF = patients off medications, PT-ON = patients on medications, PT-TOT = all patients. *=*P* < 0.05.

No significant differences were found in CMAP amplitude, distal motor latency, H-reflex latency, minimum F-wave latency, persistence, or amplitude from the APB and FCR muscles across the groups. However, APB LLRII amplitude was significantly higher in the PT-TOT group compared to HCs (96.2 µV [59.4, 157] versus 61.5 µV [40, 91.1], *P* = 0.01), mainly driven by the PT-OFF subgroup, which exhibited the highest median LLRII amplitude (105 µV [62.7, 147], *P* = 0.02) (see Fig. 3A). This difference remained significant even after normalizing LLRs amplitudes by CMAP amplitudes (LLRII/CMAP) to control the potential variability in CMAP size. Normalization further revealed a significant increase in LLRII amplitude in the PT-ON group compared to HCs (*P* = 0.03). Similarly, the FCR LLRIII amplitude was significantly higher in the PT-TOT group than in HCs (16.9 µV [11.48, 19.23] versus 10.7 µV [6.9, 16.4], *P* = 0.02), despite the relatively small sample size (see Fig. 3B). For further details and representative LLR traces, refer to Table 2 and Fig. 3C.

No significant differences were observed across subgroups for the RIII reflex (see Table 2).

### Axonal excitability

**Figure 4:**
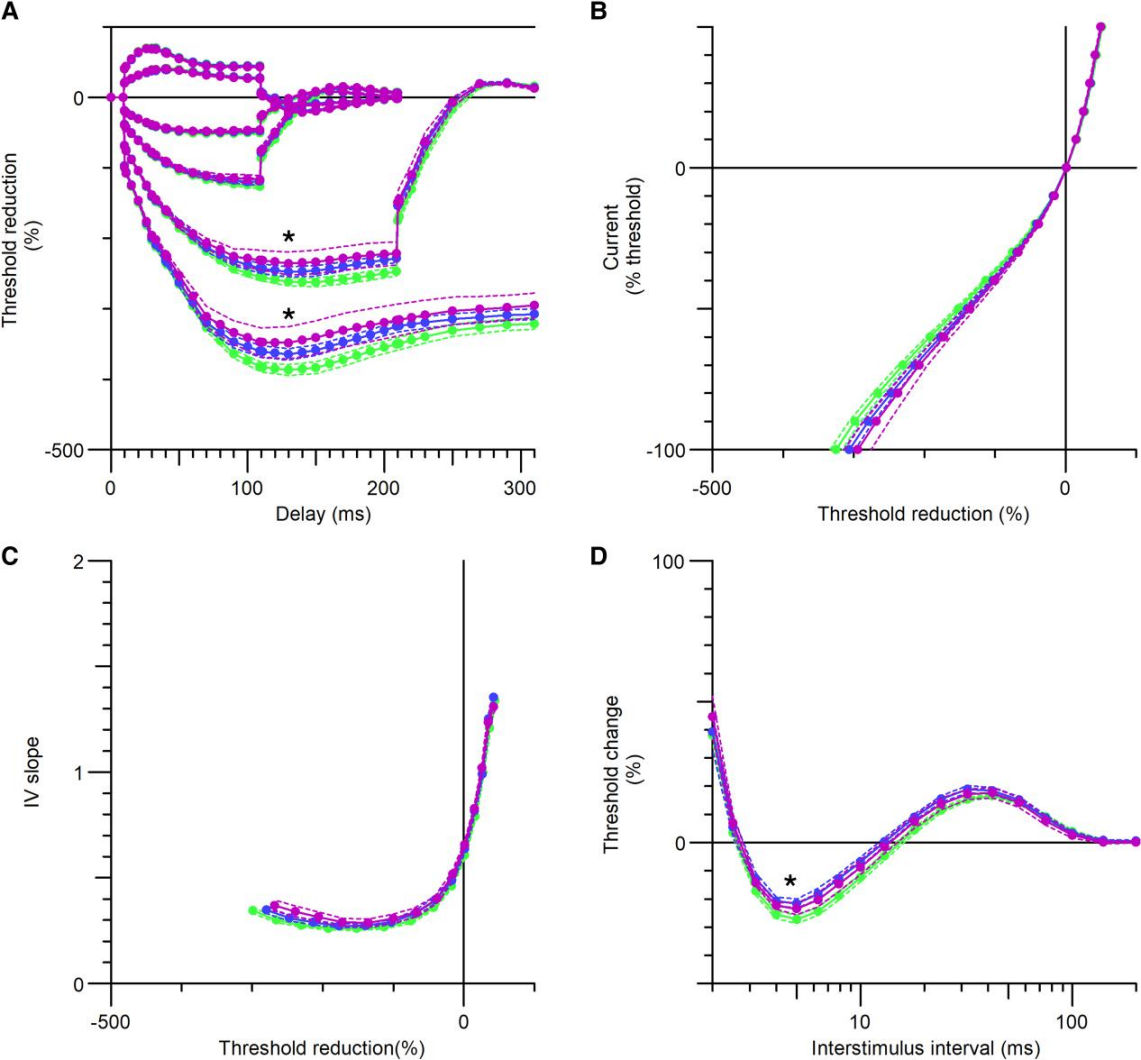
**Axonal excitability in patients with restless limbs syndrome (RLS), stratified by medication status.** (**A**) Threshold electrotonus (TE) curves with 20% and 40% depolarizing stimuli and 20%, 40%, 70% and 100% hyperpolarizing stimuli showed significant differences between PT-OFF and PT-ON in TEh[peak, −70%] and TEh[peak, −100%]. Each data point represents the mean threshold reduction for each group at a specific ISI. (**B**) Current–voltage (*I*/*V*) relationship. Each data point represents the mean threshold reduction following either a depolarizing or a hyperpolarizing stimulus, progressing stepwise from +50% (depolarizing) to −100% (hyperpolarizing) for each group. (**C**) *I*/*V* slope, representing the threshold analogue of conductance as a function of membrane potential. Each data point represents the change in *I*/*V* slope following either a depolarizing or a hyperpolarizing stimulus, applied stepwise from +50% to −100% for each group. (**D**) Recovery cycle showed a statistically significant reduction in superexcitability in PT-ON compared to PT-OFF. Each point represents the threshold change following a supramaximal stimulation at a given ISI. See text for detailed interpretation. Student’s *t*-test was applied to normally distributed variables and Mann–Whitney U test to non-normally distributed variables. Green: patients without medication (PT-OFF, *N* = 25), blue: patients on low medication doses (×<3 starting dose, PT-low, *N* = 14), magenta: patients on medium-to-high medication doses (×>3 starting dose, PT med-high, *N* = 11). ISI = inter-stimulus interval. LEDD = L-dopa equivalent daily dose. *=*P* < 0.05.

**Figure 5:**
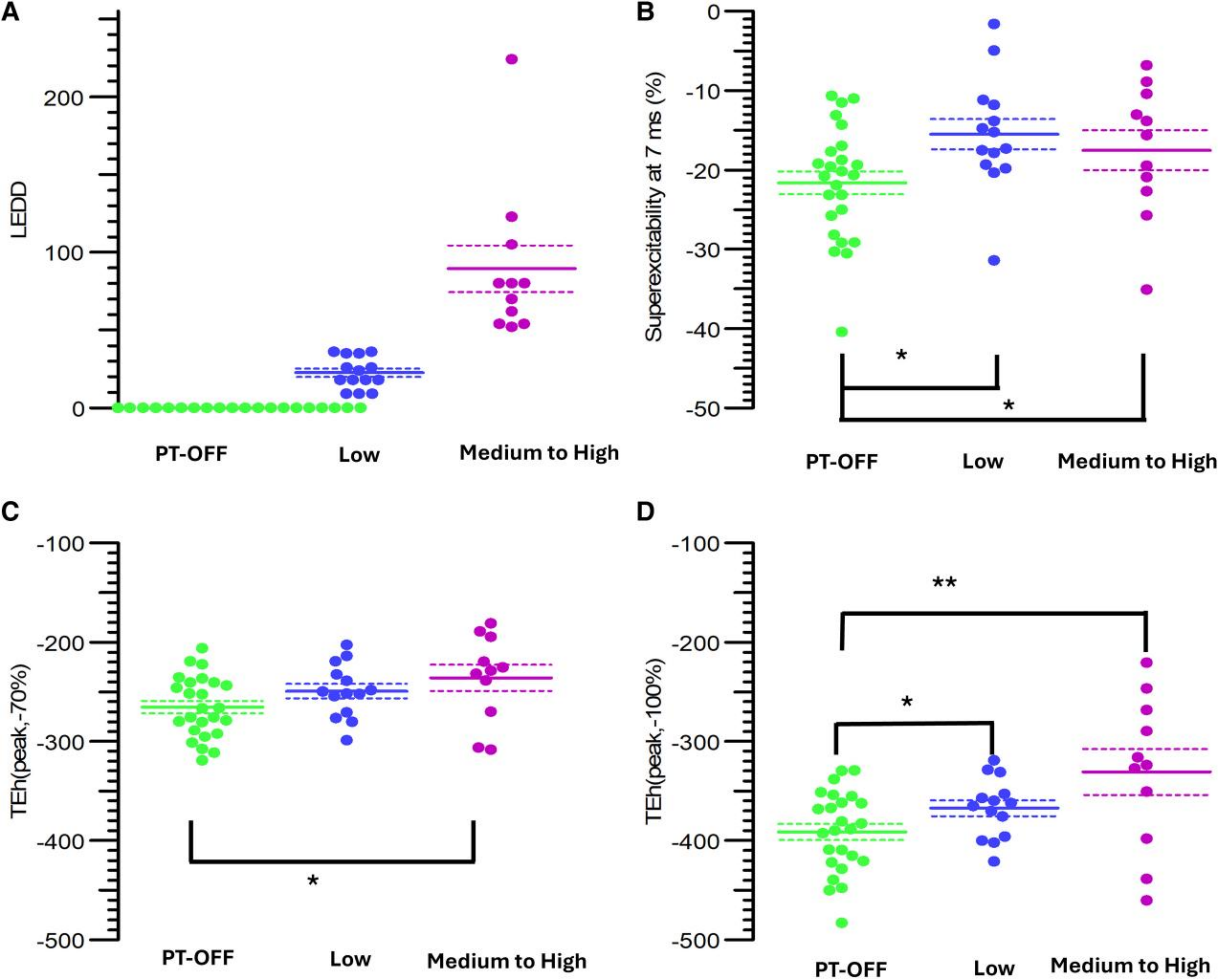
**LEDD cutoffs and key axonal excitability differences.** (**A**) LEDD values across the three patient groups: PT-OFF medications, PT-low and PT medium-to-high medication doses. (**B**) Superexcitability at 7 ms for the same groups; each dot represents an individual patient’s superexcitability value. (**C**) Threshold electrotonus differences following a hyperpolarizing conditioning stimulus at 70% of the calculated threshold (TEh(-70%)). Each dot represents one patient. (**D**) Threshold electrotonus differences following a hyperpolarizing conditioning stimulus at 100% of the calculated threshold (TEh(-100%)). Each dot represents one patient. See text for detailed interpretation. For all panels, individual data points are displayed, and central bars with dashed lines represent mean ± standard error (SE). Student’s *t*-test was applied to normally distributed variables and Mann–Whitney U test to non-normally distributed variables. Green: patients without medication (PT-OFF, *N* = 25), blue: patients on low medication doses (×<3 starting dose, Low, *N* = 14), magenta: patients on medium-to-high medication doses (×>3 starting dose, Medium-to-high, *N* = 11). LEDD = L-dopa equivalent daily dose. *=*P* < 0.05, **=*P* < 0.05.

The principal differences in axonal excitability between patients and HCs are summarized in Figs 4 and 5. Figure 4 shows a progressive increase in accommodation to hyperpolarization across groups, from PT-OFF to PT-ON low and PT-ON med-high. This trend was particularly evident using strong hyperpolarization currents (TEh[peak, −100%], *P* < 0.005; TEh[peak, −70%], *P* = 0.044). A modest reduction in superexcitability was also in the PT-ON compared to PT-OFF; however, this difference is likely attributable to age, as the PT-ON were older (*P* = 0.04) and previous studies have demonstrated that superexcitability tends to decrease with age.^77-79^

## Discussion

TMS revealed significantly reduced SICI and LICI in patients, with a more pronounced reduction observed in the treated cohort. Patients also exhibited increased APB-LLRII amplitude, while no significant differences were observed in RIII parameters. Axonal excitability tests indicated an increase in hyperpolarization-activated currents in the cohort of patients receiving the highest medication dose before the study, presumably representing the most severely affected subgroup.

Overall, our data strongly suggest reduced cortical inhibition in M1_hand_, indicating that the pathological process of altered excitability extends beyond M1_leg_ and may account for the ‘restless hands’ reported by some patients in clinical practice. Although none of the participants in our cohort reported ‘restless hands’ symptoms, our findings may suggest subclinical involvement. These results are consistent with previous literature.^32,34-36,39^ However, the more marked reduction in cortical inhibition among patients taking dopamine agonists contrasts with some earlier studies. For instance, Nardone *et al.* examined 14 patients before and after four weeks of cabergoline treatment and reported normalization of previously altered SICI following therapy.^34^ Unlike their study, which involved drug-naïve patients with variable symptom severity, our PT-ON patients had been on medication for many years. We hypothesize that patients with more severe symptoms (and therefore greater alteration in excitability) are more likely to seek treatment, possibly explaining the greater SICI impairment in the PT-ON group. Conversely, those with milder symptoms may not have sought treatment (PT-OFF) or may have been able to discontinue it prior to the study (being therefore included in PT-OFF). This suggests that SICI alterations might exist on a spectrum, from pseudo-normal in mild cases to paradoxical facilitation in severe cases, as proposed by Magalhaes *et al.*^36^ Additionally, SICI impairment in the PT-ON group might have been even more pronounced before treatment initiation. Importantly, the hypothesis that dopamine-agonist therapy induces SICI alterations can be ruled out, as these medications are known to increase SICI rather than decrease SICI,^21,34,35,80^ and SICI changes were also evident in the PT-OFF group.

Regarding LICI, its alteration in the PT-ON subgroup could be related to the augmentation phenomenon, as suggested by Salas *et al.,*^37^ or reflect another disease-related change in cortical excitability. Notably, LICI abnormalities were observed exclusively in medicated patients, likely those with more severe disease; however, since we did not systematically assess augmentation, definitive conclusions cannot be drawn.

Despite exploring a wide range of ISIs, we found no significant changes in SICF or ICF. Since this is the first study to investigate SICF in this context, comparisons with prior research are not possible. Our ICF findings align with some studies^34,37^ but contrast with others,^32,39^ indicating that further research is needed to clarify these discrepancies.

Regarding CSP, no significant differences were observed between patients and healthy controls. This is consistent with some prior reports,^39,46,47^ though it contrast with others.^38,44,45^ A recent study by De Paiva and colleagues highlighted substantial variability in CSP duration depending on the analysis method used, underscoring the importance of standardized CSP definitions and detailed methodological reporting to enable better cross-study comparisons.^47^ Their results also showed no significant differences in CSP duration between RLS patients and controls, further suggesting that CSP duration in hand muscles is unlikely to serve as a reliable biomarker for RLS.

In terms of LLRs, the latencies obtained in our cohort were consistent with those reported in a recent methodological study.^81^ In our cohort, we found a significant increase in LLR amplitude in patients compared with healthy controls, which may reflect cortico-subcortical hyperexcitability associated with RLS. Specifically, changes in LLRII and LLRIII suggest disrupted central sensory-motor integration, potentially linked to dopamine deficiency.^63,65,82-84^ Taken together, our findings support the notion that RLS is characterized by abnormal sensory-motor integration at both cortical and subcortical level. These abnormalities may be influenced by altered subcortical projections and peripheral afferent input.^50,85-87^ The disruption appears to involve not only the primary and secondary somatosensory cortices but also the basal ganglia-thalamocortical loop.^85-87^ A recent multimodal MRI study identified progressive white matter degeneration in key somatosensory circuits, which may underlie the characteristic sensory discomfort experienced in the legs by RLS patients.^88^

In our assessment of spinal excitability, we did not observe significant alterations in the RIII reflex. When considered alongside previous studies, reporting increased RIII amplitude in the lower limbs,^11^ this may indicate that spinal cord excitability abnormalities are more pronounced in the lower limbs rather than upper limbs. One possible explanation lies in the anatomical difference in pathway length: the supraspinal projections to the lumbar spinal cord are longer than those to the cervical cord, potentially making them more susceptible to impaired supraspinal inhibition. This could help explain why RLS symptoms typically present in the legs rather than the arms. Nonetheless, subtle spinal cord alterations in the upper limbs may have gone undetected in our study due to methodological or sensitivity limitations.

With regard to axonal excitability, our findings align with earlier research demonstrating increased hyperpolarization-activated currents in unmedicated patients. These results support the hypothesis that such currents may contribute to symptom exacerbation or represent an underlying predisposition to RLS. While current evidence does not establish a direct relationship between axonal excitability and SICI/LICI measures, the possibility of indirect interactions cannot be entirely discounted.

**Figure 6:**
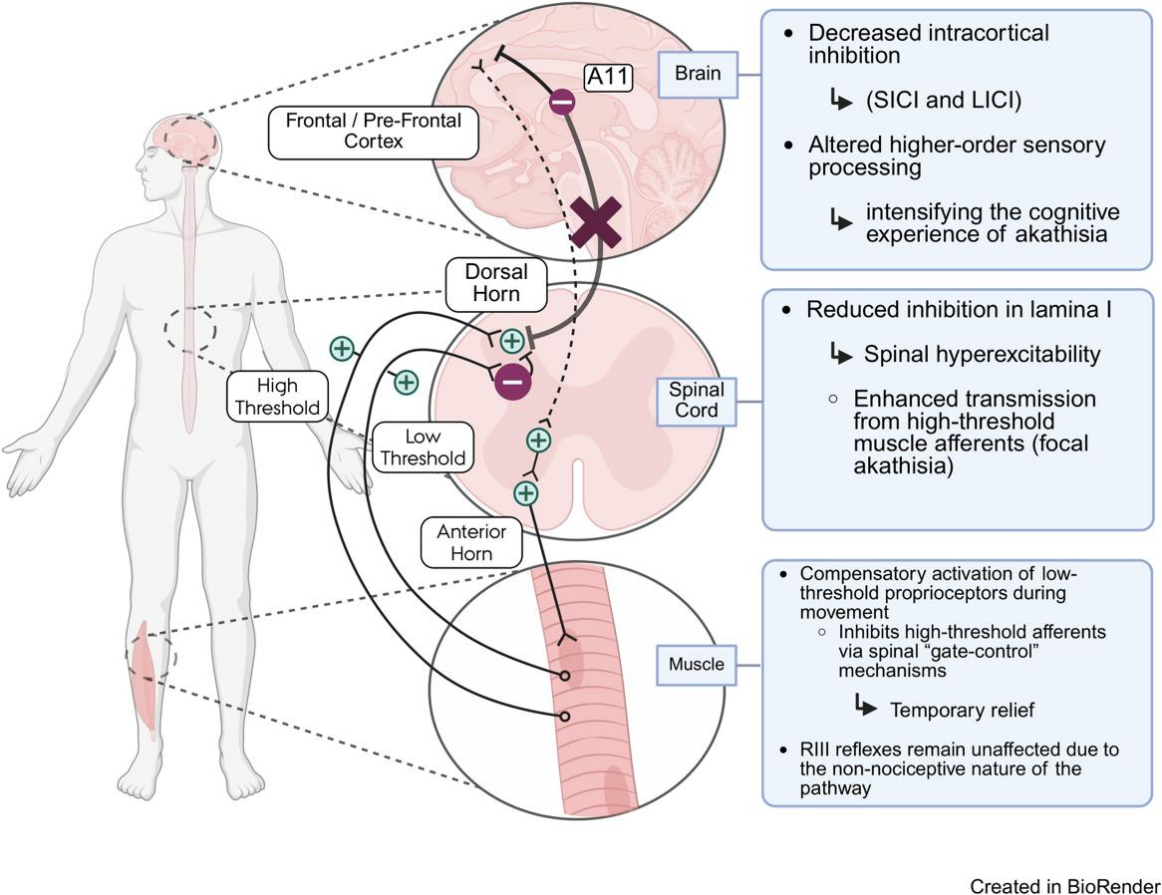
**Schematic representation of how dopamine-mediated pathways from the A11 cell group in the dorsoposterior hypothalamus may modulate cortical and spinal excitability.** In rodents, A11 neurons influence both cortical and spinal circuits, with dopaminergic tone exhibiting a circadian pattern (lowest at night). Based on existing literature and our results, we propose that nocturnal reduction of A11 input: (1) Cortex: may contribute to decreased intracortical inhibition (SICI and LICI) and altered higher-order sensory processing, potentially intensifying the cognitive experience of akathisia. (2) Spinal cord: may reduce inhibition in lamina I (red cross), resulting in spinal hyperexcitability and enhanced transmission from high-threshold muscle afferents (focal akathisia). (3) Muscle: may trigger compensatory activation of low-threshold proprioceptors during movement, which inhibit high-threshold afferents via spinal ‘gate-control’ mechanisms, offering temporary relief. RIII reflexes remain unaffected due to the non-nociceptive nature of the pathway. A11 = group of neuron cells located in the dorsoposterior hypothalamus. LICI = long-interval intracortical inhibition, SICI = short-interval intracortical inhibition. Created with BioRender. Available at: https://BioRender.com/ed6hy3l.

An intriguing hypothesis that integrates clinical observations, existing literature, and our current findings is illustrated in Fig. 6. It proposes that supraspinal sensory-motor circuits, including dopaminergic pathways originating from the A11 cell group in the dorsoposterior hypothalamus, modulate spinal reflex excitability.^48,89^ Preclinical studies suggest that dopaminergic system projects not only to the spinal cord but also to frontal and prefrontal cortical regions in rodents, potentially influencing both cortical and spinal excitability.^90^ A circadian decline in dopaminergic tone from the A11 nucleus^91^ may weaken inhibitory control over both the motor cortex and lamina I of the spinal cord, the latter of which relays high-threshold deep afferent inputs to the brain and expresses D1-like and D2-like receptors.^89^ Supporting this, descending inhibition via autogenic axo-axonal presynaptic mechanisms has also been described.^92^ The loss of dopaminergic input may thus lead to spinal segmental hyperexcitability and heightened transmission of signals from high-threshold muscle afferent, particularly those originating in the lower limbs. Combined with the abnormal sensory-motor integration indicated by LLRs, this may explain the ‘urge to move the limbs’ experienced by RLS patients. Voluntary movement activates proprioceptive pathways that do not encode pain and can inhibit high-threshold afferent signalling via a spinal ‘gate-control’ mechanism, providing transient relief of symptoms.^89,93,94^

The observed abnormalities in LLRII and LLRIII may therefore share a common pathophysiological basis with impaired descending dopaminergic modulation from the A11 system. This is further supported by the significant differences in LLRs between unmedicated patients and healthy controls, suggesting that dopaminergic treatment partially modulates abnormal sensory-motor integration and contributes to symptom relief. Conversely, the absence of RIII reflex changes in upper limb muscles reinforces the idea that regional spinal cord alterations are necessary for symptom manifestation and that nociceptive pathways are not primarily involved in the disorder.

In healthy individuals, the physiological nocturnal decline in dopaminergic tone appears insufficient to trigger symptoms. In contrast, in patients the dopaminergic tone may fall below a critical threshold during the night, triggering the emergence of symptoms.

## Conclusion, limitations and future directions

This study represents the largest neurophysiological investigation of RLS to date, providing robust and comprehensive data that enhances the generalizability of the findings. Importantly, it is the first to assess SICF in this condition, alongside with a broad range of ISIs for SICI, ICF and LICI using threshold-tracking TMS.

Our results support the hypothesis that RLS pathophysiology is characterized by reduced motor cortex inhibition and disrupted sensory-motor integration. However, given that most examinations were conducted in the morning, when symptoms are typically minimal, cortical and spinal abnormalities may have been even underestimated.

The study also highlights the potential therapeutic relevance of targeting hyperpolarization-activated currents, suggesting a novel and promising avenue for RLS treatment.

One notable limitation is the absence of a standardized clinical scale to assess disease severity. Instead, we used pre-study dopaminergic medication dosage as a surrogate marker, which may not accurately reflect clinical symptom burden. This limitation underscores the need for future studies incorporating standardized severity measures.

Further research is warranted to elucidate why spinal cord hyperexcitability appears to predominantly, if not exclusively, affect the lower limbs, to clarify the functional significance of altered axonal excitability and to determine how neurophysiological parameters evolve in individual patients before and after medication administration.

## Supplementary Material

fcaf506_Supplementary_Data

## Data Availability

Data are available by contacting the Corresponding Author.
