# Supplementary material for: Central and peripheral excitability in restless limbs syndrome
Source: Brain Commun. 2025 Dec 24;8(1):fcaf506. doi: 10.1093/braincomms/fcaf506 (PMC12784252; doi:10.1093/braincomms/fcaf506)
Supplement: fcaf506_Supplementary_Data [file fcaf506_supplementary_data.docx]

**SUPPLEMENTARY MATERIAL**


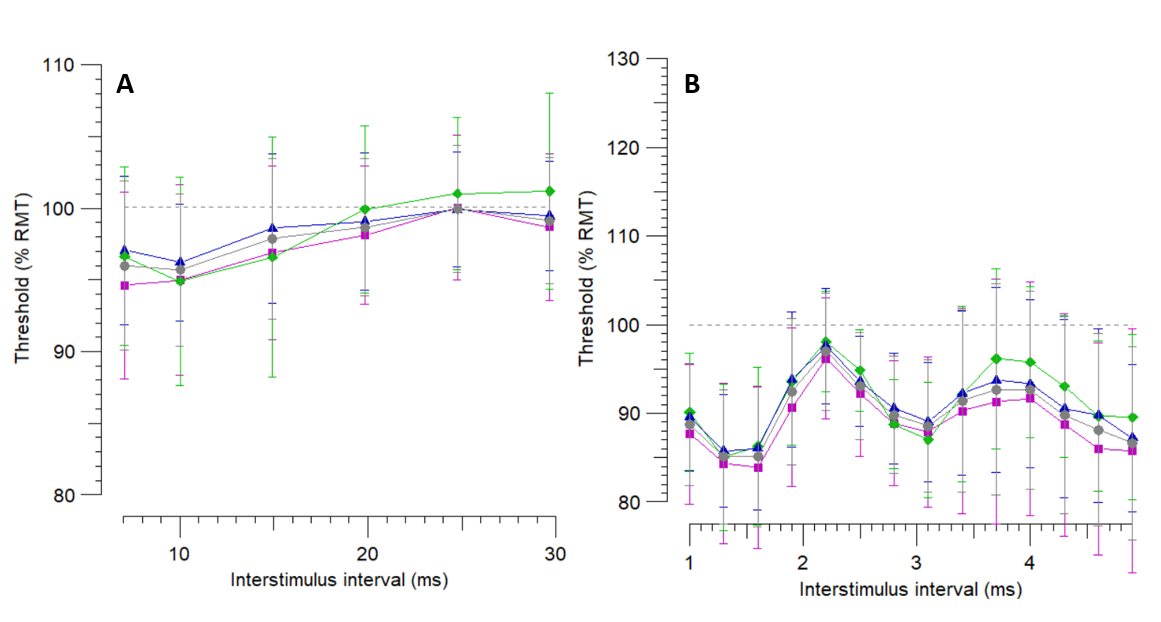


**Supplementary Figure 1.** Parallel threshold-tracking intracortical facilitation (ICF, A) and short-interval intracortical facilitation (SICF, B) recorded at interstimulus intervals (ISIs) of 7–30 ms and 1–4.9 ms, respectively. Larger downward deflections indicate greater facilitation. Each point represents a single ISI. Data are shown as mean ± SD. No statistically significant differences were observed between groups at any ISI. Student’s t-test was used. Magenta squares: patients on medication (PT-ON, *N*=24); green diamonds: healthy controls (HC, *N*=32); blue triangles: patients off medication (PT-OFF, *N*=32); grey dots: all patients (PT-TOT, *N*=56). ISI = interstimulus interval, RMT = Resting motor threshold.
